# Temporal Kinetics of Circulating Oxidative Stress Markers in Intracerebral Hemorrhage and Their Association with Perihematoma Edema

**DOI:** 10.3390/antiox15101260

**Published:** 2026-10-01

**Authors:** Sandeep Kumar Gupta, Jayantee Kalita, Prakash C. Pandey, Dhiraj Kumar, Vivek Singh, Roopali Mahajan

**Affiliations:** 1 Department of Neurology, Sanjay Gandhi Post Graduate Institute of Medical Sciences, Raebareli Road, Lucknow 226014, Uttar Pradesh, India; sandeepngci@gmail.com (S.K.G.);; 2 Department of Neurology, Baba Raghav Das Medical College, Gorakhpur 273013, Uttar Pradesh, India; 3 Department of Radio Diagnosis, Sanjay Gandhi Post Graduate Institute of Medical Sciences, Raebareli Road, Lucknow 226014, Uttar Pradesh, India

**Keywords:** intracerebral hemorrhage, antioxidant, oxidative stress, perihematoma-edema, outcome

## Abstract

In intracerebral hemorrhage (ICH), an elevated oxidant level may lead to perihematoma edema (PHE) and adversely affect its outcome. We report circulating oxidative stress markers in ICH patients at different time points and their association with PHE on computerized tomographic (CT) scans. A total of 87 patients with CT confirmed ICH within 24 h of ictus were prospectively included, and a follow-up CT scan was performed on day 7. The location and volume of hematoma, hematoma edema complex (HEC), PHE, and intraventricular extension of hemorrhage were noted on both CT scans. Blood samples were collected on days 1, 7, and 15, and reactive oxygen species (ROS), malondialdehyde (MDA), catalase (CAT), and glutathione peroxidase (GPx) levels were measured using a microplate reader. These biomarkers were also measured in 57 age-matched healthy controls. The patients had elevated ROS (*p* < 0.001) and MDA (*p* < 0.001) levels, whereas CAT (*p* < 0.001) and GPx (*p* < 0.001) levels were reduced compared to the controls. The oxidants remained elevated on day 7 and decreased on day 15 but did not reach the control levels. Furthermore, the CAT and GPx showed recovery on day 7 and day 15 but did not achieve the control levels. ROS (r = 0.28, *p* = 0.02) and GPx (r = −0.29, *p* = 0.02) correlated with PHE, suggesting their role in the pathogenesis. The hematoma volume, HEC, and PHE at day 1 correlated with the day 15 CAT level and MDA with PHE. At one month, 15 (17.24%) patients died, 56 (64.36%) had poor recovery, and 16 (18.39%) had good recovery. The patients with higher antioxidant levels had better survival and good outcomes. Future studies may investigate the efficacy of antioxidant therapy in improving clinical outcomes and modulating oxidative stress biomarkers in ICH patients.

## 1. Introduction

Stroke is the second leading cause of death and the third leading cause of mortality and disability combined globally [1]. In 2021, about 11.9 million incident cases and 93.8 million prevalent stroke cases were estimated, resulting in approximately 7.3 million deaths [2]. Ischemic stroke is the most common type, accounting for nearly 80–90% of cases, while intracerebral hemorrhage (ICH) constitutes about 10–20% of stroke cases [3,4]. Hemorrhagic stroke is notably more prevalent in Asia, accounting for 20–30% of reported cases [5,6].

Post-stroke cerebral edema is an important determinant of clinical worsening, mortality and functional outcome [7]. On the basis of pathophysiology, cerebral edema can be categorized as cytotoxic, vasogenic, interstitial, osmotic, or combined. Cytotoxic edema occurs in arterial infarction and small vessel disease; vasogenic edema occurs in ICH, brain tumors, and cerebral venous thrombosis; interstitial edema occurs in hydrocephalus; osmotic edema occurs with rapid lowering of serum osmolality; and a combination of cytotoxic and vasogenic edema may occur in stroke, tumor, and infections [8]. The outcomes of ICH depend not only on the primary brain damage caused by the hematoma but may also depend on the secondary brain injury induced by hemoglobin breakdown products such as hemin and iron. Hemin (ferric iron-protoporphyrin IX) and its constituent iron play a dual role in generating reactive oxygen species (ROS), acting as a direct catalyst for free radical production as well as a substrate for intracellular, pro-oxidant reactions. Iron promotes Fenton chemistry and may sustain Haber–Weiss-type redox cycling, generating highly reactive hydroxyl radicals (•OH). These radicals may trigger lipid peroxidation-mediated cell death, dysregulate autophagy and oxidize nucleic acid; therefore, they may promote secondary brain injury and neurodegeneration following ICH [9,10,11,12,13,14]. The brain is more vulnerable to oxidative injury due to its rich lipid and iron content, as well as the relative deficiency of glutathione peroxidase (GPx), superoxide dismutase (SOD), and nuclear factor erythroid-2-related factor 2 (Nrf-2) [10,15]. Both animal and human studies have shown increased oxidants and reduced antioxidants during the acute stage of stroke [16]. A collagenase-induced rodent ICH study has reported a 2.1-fold higher level of oxidation protein in the perihematomal region than in the contralateral striatum. Moreover, there are elevated levels of nitrosylated and carbonylated proteins and matrix metallopeptidase-9 (MMP-9) [10,17,18]. These findings indicate that a redox-imbalanced environment surrounding the hematoma may cause endothelial dysfunction and blood–brain barrier disruption, exacerbating perihematomal edema (PHE) and worsening patient outcomes.

In principle, disease biomarkers are measured in the target tissue/organs; however, direct assessment of oxidative stress biomarkers in the central nervous system (CNS) is often not feasible in clinical studies [16]. Because most reactive oxygen species (ROS) are highly unstable and short-lived, those generated within brain tissue are rarely detected directly in peripheral blood. However, an acute CNS insult may trigger a surge in catecholamines, inducing systemic oxidative stress. Consequently, circulating biomarkers of oxidative stress can serve as valuable indirect indicators of CNS pathology [16,19,20].

Clinical studies investigating oxidative stress biomarkers in ICH patients are limited; a PubMed search on 12 September 2026, using the keywords ‘intracerebral hemorrhage’ and ‘oxidative stress’, identified only 12 relevant human studies [21,22,23,24,25,26]. The majority of these studies reported elevated markers of circulating oxidative stress and reduced levels of antioxidants [27,28,29,30]; five studies correlated the findings with radiological findings (CT/MRI) [27,28,29,30,31] and three with death [28,29,30]. However, one study evaluated the temporal kinetics of oxidants and antioxidants in ICH patients [32] and none evaluated the association of oxidative stress with day 1 and day 7 PHE. Based on this knowledge gap, we hypothesized that elevated oxidants may result in PHE, and these patients may experience higher mortality and poorer outcomes at one month. In this communication, we report the temporal kinetics of circulating oxidative stress markers [ROS and malondialdehyde (MDA)] and antioxidants [catalase (CAT) and GPx] on days 1, 7, and 15 of ICH and the influence of these biomarkers on the hematoma edema complex (HEC) and PHE on a repeat CT scan on day 7.

## 2. Subjects and Methods

This is a prospective observational single-center hospital-based study of a cohort of patients with ICH admitted to our care between January 2023 and December 2024. The study protocol was approved by the Ethics Committee of Sanjay Gandhi Postgraduate Institute of Medical Sciences, Lucknow, India (Ethics No. 2022-20-IMP-125, 8 July 2022). Eligible patients or their guardians gave informed consent.

### 2.1. Inclusion Criteria

Patients admitted within 24 h of the acute onset of a neurological deficit with or without headache, vomiting, altered sensorium, or seizure, and whose cranial CT scan revealed an ICH were included.

### 2.2. Exclusion Criteria

Patients with subarachnoid hemorrhage, subdural hematoma, vascular malformation, tumor bleed, bleeding diathesis, head injury, malignancy, anticoagulant or thrombolysis-induced ICH, or venous thrombosis; children (≤18 years), elderly (>80 years), and those with pregnancy, lactation, organ transplantation, immunosuppression, acquired immune deficiency syndrome, or did not provide consent were excluded.

### 2.3. Clinical Evaluation

A detailed history, including demographic information (age and gender) and stroke risk factors (hypertension, diabetes, smoking, obesity, and sedentary habit) was recorded. The presenting symptoms, including hemiplegia, quadriplegia, visual abnormalities, seizures, headaches, vomiting, and alterations in consciousness were noted. Consciousness was assessed using the Glasgow Coma Scale (GCS), and stroke severity was assessed using the National Institute of Health Stroke Scale (NIHSS).

### 2.4. Investigations

Hemoglobin, blood counts, erythrocyte sedimentation rate at the first hour, blood glucose, serum creatinine, sodium, potassium, albumin, calcium, and alkaline phosphatase were measured. Electrocardiography and chest radiography were performed.

### 2.5. Computerized CT Scan

A cranial CT scan was done using a 64-slice CT scanner (Philips Brilliance 64-slice, Philips Medical System, Eindhoven, The Netherlands), and the location and volume of the hematoma, the midline shift, and intraventricular extension were recorded. A repeat CT scan was done on the 7th day. The hematoma volume, HEC, and absolute PHE were measured using Philips IntelliSpace Portal Version 12.1, in which volumetric analysis was done by clip and 3D segmentation. A hand-drawn ROI (region of interest)-based method was used to outline the hematoma and the PHE in each slice, and an automated software calculated the volume.

### 2.6. Biomarker Assay

Five milliliters of venous blood from the antecubital vein were drawn in an ethylenediamine tetra-acetic acid and plain vials on the 1st, 7th, and 15th days of stroke. Plasma and Serum were separated from the respective samples and kept at −80 °C until analyzed. The following oxidants and antioxidants were measured.

Reactive Oxygen Species: A 25 µL aliquot of serum was added in triplicate to each well of the 96-well plate along with a blank. The volume was made up by adding 174 µL of 1x phosphate-buffer (pH = 7.4) along with 1 µL of 2,7-dichlorofluorescein diacetate (DCFH) dye (Cat. No. 85048, from Sisco Research Laboratories Private Limited, Mumbai, Maharashtra, India) to achieve a final volume of 200 µL in each well. The plate was then incubated at 20 °C in the dark on an orbital shaker for 30 min. The fluorescence intensity was measured at 485 nm for excitation and 535 nm for emission using a microplate reader (Synergy H1, BioTek Instruments Inc., Winooski, VT, USA) [33].

Malondialdehyde: A 25 µL aliquot of serum was mixed with 825 µL of distilled water in a test tube, followed by brief vortexing for 5 s. A 50 µL aliquot of 10% sodium dodecyl sulfate was added, and the mixture was allowed to incubate for 5 min at room temperature. Next, 300 µL of 20% glacial acetic acid (GAA) (Cat. No. 1.93402.0521, from Merck Life Science Private Limited, Mumbai, India) was added and incubated for 5 min. 300 µL of 0.8% thiobarbituric acid (TBA) (Cat. No. AST2873, Avra Synthesis Private Limited, Hyderabad, India) solution was added, and the mixture was incubated in a water bath at 95 °C for an hour, during which the color of the sample changed from colorless to pink. The samples were then cooled at 4 °C for 15 min and centrifuged at 5000 rpm for 5 min. The pellet was discarded, and the supernatant was transferred to each well of a 96-well plate. The absorbance was measured at 532 nm using a microplate reader [34,35].

Catalase: Four tubes were labeled as experimental test, control test, standard, and blank, respectively. A 1 mL aliquot of 0.01 M sodium phosphate buffer was added in each tube, and 100 µL of serum (1 mg/mL protein) was added to the experimental and control test tubes only. After that, distilled water was added as follows: 1000 µL to the control tube, 100 µL to the standard, and 1100 µL to the blank tube. A 1000 µL aliquot of 0.4 M H_2_O_2_ (Cat. No. Q18755, from Thermo Fisher Scientific India Private Limited, Mumbai, India) was added to the experimental and standard tubes, respectively. The samples were briefly vortexed and incubated at 37 °C for 3 min, followed by the addition of 2 mL of 5% dichromate acetic acid (DAA) to each of the four tubes. All the tubes were incubated at 100 °C for 10 min. The tubes were allowed to cool under running tap water and centrifuged at 2500× *g* for 5 min to separate the precipitated protein. The absorbance was measured at 470 nm using a microplate reader [36,37].

Glutathione peroxidase: Aliquots of 60 µL of 0.1 M phosphate buffer (pH = 7.4), 40 µL of 2 mM reduced glutathione (GSH) (Cat No. GLR09.022149, GLR Innovations, New Delhi, India), 20 µL of 10 mM sodium azide (NaN_3_) (Cat. No. 84070, Sisco Research Laboratories Private Limited, Mumbai, Maharashtra, India), and 20 µL of 1 mM H_2_O_2_ were added to 60 µL of plasma sample in a microcentrifuge tube, followed by brief mixing, and allowed to incubate for 15 min at 37 °C. A 100 µL aliquot of 5% trichloroacetic acid (TCA) (Cat No. GLRIN19088672, GLR Innovations, New Delhi, India) was added, then the mixture was centrifuged at 2000 rpm for 5 min. After that, 25 µL of supernatant was transferred to a new microcentrifuge tube with 35 µL of 0.1 M phosphate buffer, and 140 µL of 0.4 mg/mL 5,5-dithionitrobenzoic acid (DTNB) (Cat No. GLRIN19013648, GLR Innovations, New Delhi, India) was further added after vortexing for 30 s. The color of the sample changed from colorless to yellow. The sample was transferred to each well of the 96-well plate. Absorbance was measured at 420 nm using a microplate reader [38].

### 2.7. Control Group

Fifty-seven healthy age- [56 (Q1, 51–Q3, 59) vs. 58 (Q1, 50–Q3, 68) years, *p* = 0.10] and gender-matched [females 18 (30.5%) vs. 33 (37.9%), *p* = 0.38] controls were recruited from among hospital employee or patients’ relatives to establish reference values for oxidative stress biomarkers. They did not have hypertension, diabetes mellitus, hyperlipidemia, or a history of stroke and were nonsmokers and nonalcoholics.

### 2.8. Treatment

All the patients were managed in the high-dependency unit (HDU) with continuous monitoring of the electrocardiogram, blood pressure, respiratory rate, and oxygen saturation. Patients who developed respiratory distress with evidence of arterial blood gas abnormalities, including hypoxia, hypercarbia, or acidosis, were intubated and mechanically ventilated. These patients were managed in the intensive care unit (ICU). Antihypertensive drugs (amlodipine, metoprolol, and hydrochlorothiazide in isolation or in combination) were prescribed if blood pressure was more than 160/100 mm Hg. Patients with clinical signs of raised intracranial pressure, such as pupillary asymmetry and hyperventilation, received 100 mL of 10% mannitol intravenously. None of the patients underwent surgical evaluation for hematoma or extra-ventricular drainage.

### 2.9. Outcome

Outcome at one month was defined on the basis of the modified Rankin Scale (mRS) and was categorized as death (mRS = 6), poor outcome (mRS > 2), and good outcome (mRS ≤ 2) [39].

### 2.10. Sample Size

Sample size was calculated using G*Power 3.1.9.7. In the literature, GPx values in patients with ICH and healthy controls were 51.6 ± 14.8 U/g Hb and 64.0 ± 21.0 U/g Hb, respectively (Cohen’s d effect size = 0.682) [30]. With an α error of 0.05, a study power of 95%, and an effect size of 0.682, the estimated sample size for each group was 57. We included 87 patients with ICH and 57 healthy controls.

### 2.11. Statistical Analysis

The normality of continuous data was verified by the Shapiro–Wilk test. The baseline biomarkers of oxidative stress and antioxidants in ICH patients and healthy controls were compared using the Mann–Whitney U test. These oxidative stress biomarkers at different time points (day 1, day 7, and day 15) were compared using the related samples Friedman’s two-way analysis of variance test with Bonferroni correction. The sizes of ICH, PHE, and HEC on day 1 and day 7 were compared using the Wilcoxon signed-rank test. The relationships of oxidants and antioxidants with clinical data, ICH, PHE, and HEC were evaluated using Spearman’s rank correlation test. The relationships of biomarkers with death, poor recovery, and good recovery were evaluated using one-way analysis of variance. The predictive value of clinical data, CT scan findings, and biomarkers in defining death and good outcome at 1 month was evaluated using univariate analysis. The variables having a two-tailed *p*-value of ≤0.05 in the univariate analysis were included in the multivariate backward-Wald logistic regression analysis with the Hosmer–Lemeshow goodness-of-fit test to derive the best set of predictors of death and good outcome. SPSS 20v was used for statistical analysis, and graphs were prepared using GraphPad Prism 7. A variable with a two-sided *p*-value of <0.05 was considered significant.

## 3. Results

**Figure 1:**
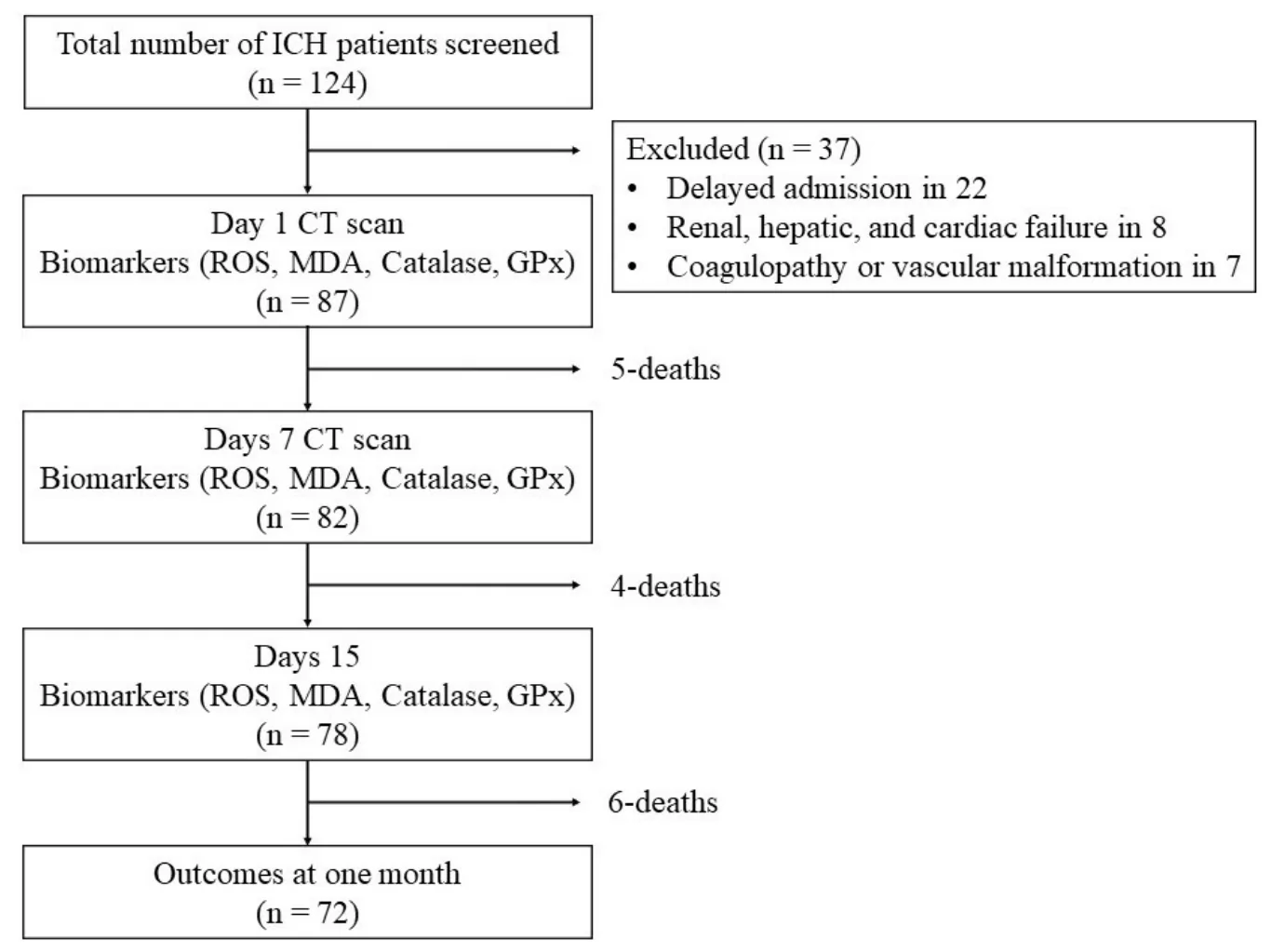
Flow chart showing the work plan.

During the study period, 124 ICH patients were admitted; 37 were excluded due to delayed admission in 22, renal, hepatic, or cardiac failure in eight, and coagulopathy or vascular malformation in seven patients. Eight of these 37 (21.6%) patients died within 2 weeks. Therefore, this study was based on 87 patients; of them, five patients died within 1 week, and another four died between 1 and 2 weeks. Hence, biomarkers could be studied in 87, 82, and 78 patients on day 1, day 7, and day 15, respectively (Figure 1). Their median age was 58 (Q1, 50–Q3, 68) years, and 33 (37.9%) were females. Seventy-nine (90.8%) patients were hypertensive, 19 (21.8%) diabetic, 11 (12.6%) smokers, 29 (33.3%) tobacco chewers, and 19 (21.8%) consumed alcohol. The median NIHSS score at admission was 18 (range 11–36), and the GCS score was 12 (ranged 3–15). The details are presented in Supplementary Table S1.

### 3.1. CT Scan Findings

**Figure 2:**
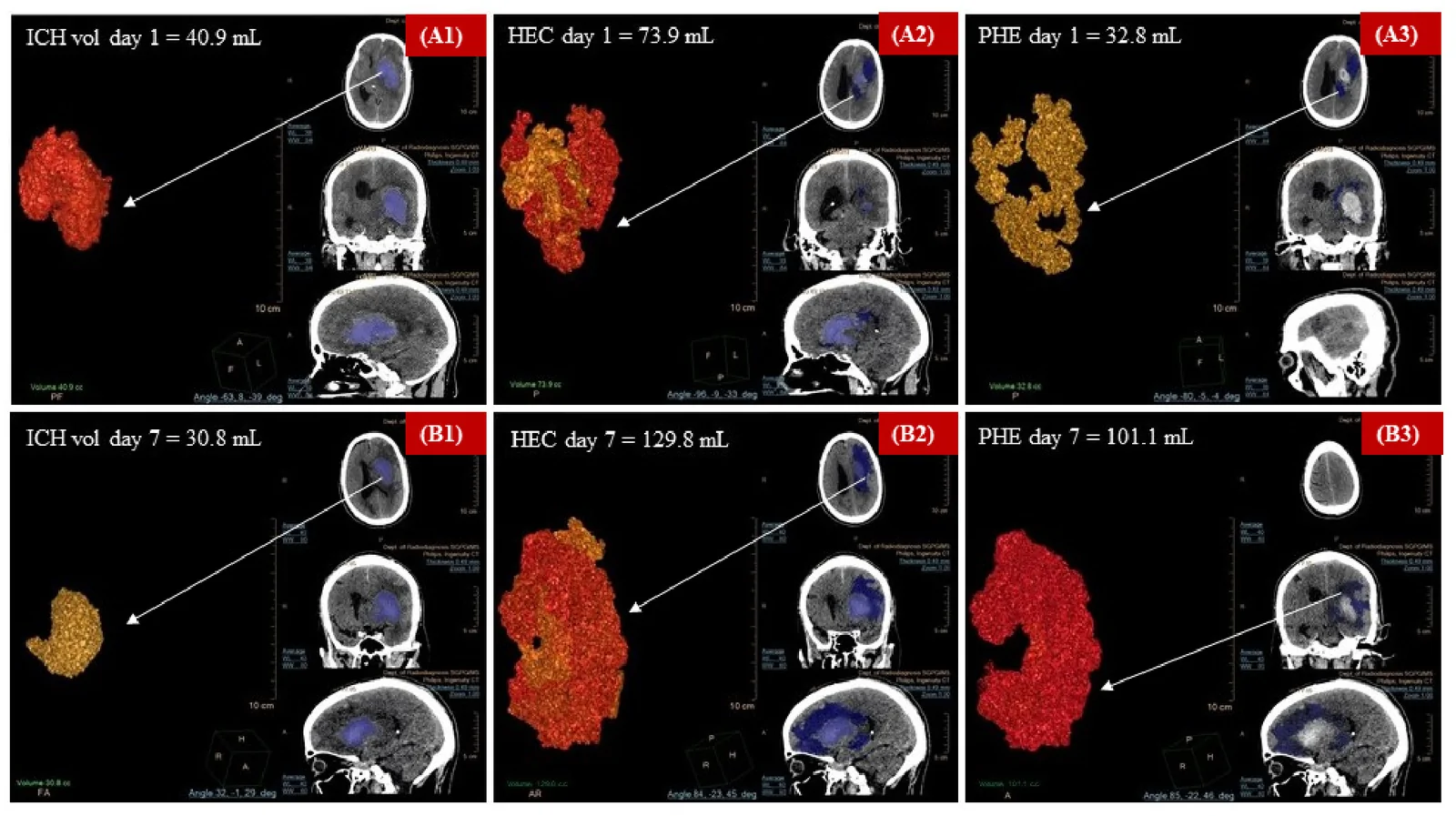
Volumetric analysis of intracerebral hematoma (ICH) on day 1 and day 7. (**A1**–**A3**) images show computerized CT scan findings: (**A1**) ICH volume, (**A2**) hematoma edema complex (HEC), and (**A3**) perihematoma edema (PHE) on day 1. (**B1**–**B3**) are the images on day 7, and show reduction in (**B1**) ICH volume, but (**B2**) HEC and (**B3**) PHE volume increased from the day 1.

Seventy-one (81.6%) patients had basal ganglia or thalamic ICH, and 16 (18.4%) had lobar ICH. On day 1 CT scan, the median hematoma volume was 25.0 (Q1, 19.0–Q3, 50.0) mL, HEC was 30.0 (Q1, 22.0–Q3, 53.5) mL, and PHE was 4.0 (Q1, 2.0–Q3, 7.0) mL; 39 (44.8%) patients had intraventricular extension. A repeat CT scan on day 7 revealed an increase in PHE (*p* < 0.001) (Figure 2). Hematoma volume and HEC, however, did not increase (Table 1).

### 3.2. Comparison of Biomarkers in the Patients and the Healthy Controls

**Figure 3:**
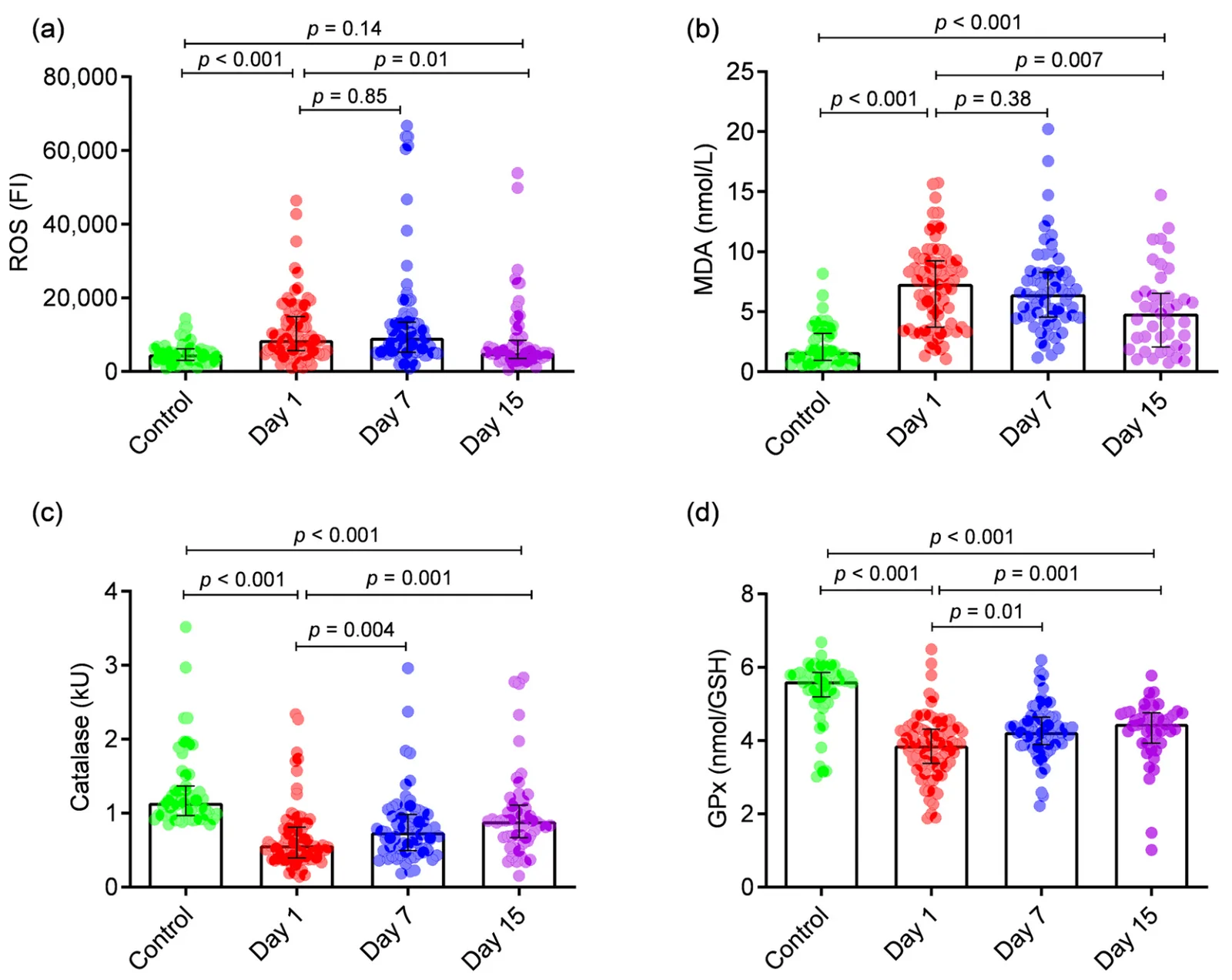
The error bar diagram shows a comparison of biomarkers in patients at different time points with normal healthy controls. (**a**) ROS (reactive oxygen species) and (**b**) MDA (malondialdehyde) levels were higher the patients compared to the controls, and remained high till day 7. On day 15, the ROS level in the patients reached that of the controls, but the MDA level remained elevated. (**c**) Catalase and (**d**) GPx (glutathione peroxidase) levels in the patients were reduced compared to the healthy controls on day 1 and recovered on day 7 and 15 but remained low compared to the controls.

On day 1, the patients had elevated ROS (*p* < 0.001) and MDA (*p* < 0.001) levels, whereas CAT (*p* < 0.001) and GPx (*p* < 0.001) were reduced compared to the controls (Table 2). There was recovery of CAT (*p* = 0.004) and GPx (*p* = 0.01) on day 7 compared to day 1. On day 15, CAT recovered further compared to day 7 (*p* = 0.012), but the recovery of GPx was not significant (*p* = 0.167). The oxidative stress markers (ROS and MDA) remained elevated on day 7 but were reduced significantly on day 15, compared to day 1. However, ROS (*p* = 0.144) and MDA (*p* < 0.001) levels remained elevated even on day 15 compared to the controls. Similarly, the patients had lower levels of GPx (*p* < 0.001) and CAT (*p* < 0.001) on day 15 compared to the controls (Table 2 and Figure 3).

### 3.3. Correlation of Biomarkers with CT Scan Findings

**Figure 4:**
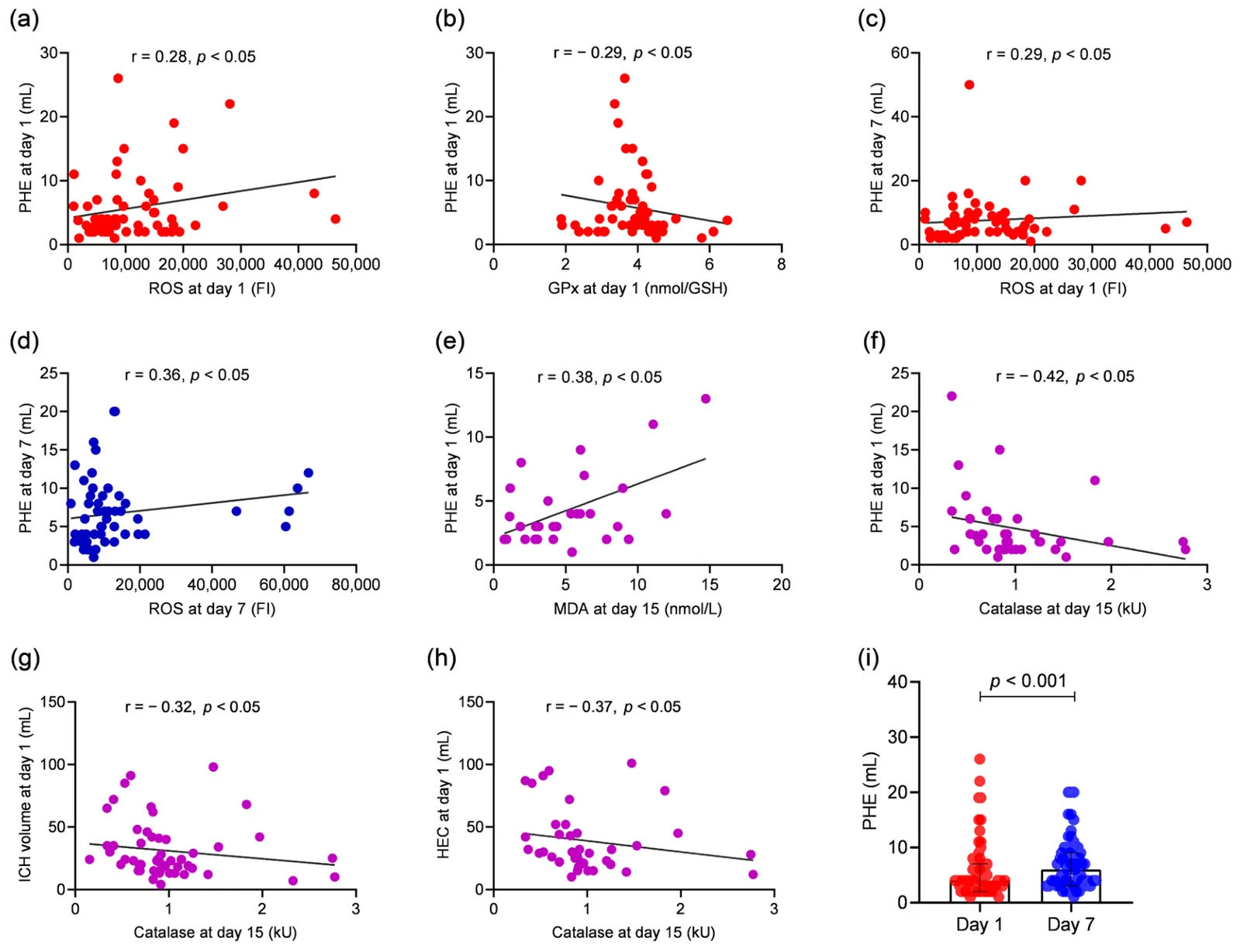
Linear regression graphs showing correlations of biomarkers with CT scan findings. Day 1 ROS (reactive oxygen species) correlated with (**a**) day 1 and (**c**) day 7 perihematoma edema (PHE). Day 7 ROS also correlated with (**d**) day 7 PHE. (**b**) Day 1 GPx (glutathione peroxidase) inversely correlated with day 1 PHE. (**e**) Day 15 MDA (malondialdehyde) correlated with day 1 PHE, and catalase correlated with (**f**) day 1 PHE, (**g**) ICH volume and (**h**) HEC (hematoma edema complex). (**i**) The error bar diagram shows an increase in PHE volume on day 7 compared to day 1. The solid black line represents the linear trendline.

The day 1 biomarkers revealed a weak to moderate correlation of ROS (r = 0.28, *p* = 0.02) and GPx (r = −0.29, *p* = 0.02) with PHE volume. There was no correlation of ICH volume and HEC with any of the oxidant or antioxidant biomarkers. On the 7th day, PHE had a moderate correlation with ROS (r = 0.36, *p* = 0.005). On day 15, the biomarkers had a moderate correlation with admission CT scan parameters: CAT with hematoma volume (r = −0.32; *p* = 0.02), PHE (r = −0.43; *p* = 0.006), and HEC (r = −0.37; *p* = 0.02), and MDA with PHE (r = 0.38; *p* = 0.04). On day 15, the biomarkers also correlated with day 7 CT scan parameters. The details are presented in Supplementary Table S2, and significant parameters are shown in Figure 4.

### 3.4. Association of Biomarkers with Outcome

At 1 month, 15 (17.24%) patients died, 56 (64.36%) had a poor outcome, and 16 (18.39%) had a good outcome. Among the biomarkers, GPx was associated with survival (Table 3). GPx levels were higher in the patients who survived compared to those who died [3.94 (Q1, 3.55–Q3, 4.36) vs. 3.30 (Q1, 2.62–Q3, 3.85) nmol/GSH, *p* = 0.02] (Table 4). Day 1 ROS and MDA levels did not show an association with the outcome. On univariate analysis, death was also associated with hematoma volume (*p* < 0.001), HEC volume (*p* < 0.001), NIHSS score (*p* < 0.001), GCS score (*p* < 0.001), GPx (*p* = 0.01), and the requirement for an antiedema drug (*p* = 0.003) and mechanical ventilation (*p* < 0.001). The requirement for antihypertensive, antidiabetic, antibiotic, and antiseizure drugs was not associated with death (Table 4). On multivariate analysis, after including all the parameters that were significant in the univariate analysis, the independent predictors of death were the NIHSS score [adjusted odds ratio (AOR) 1.18; 95% confidence interval (CI) 1.00–1.39; *p* = 0.054) and mechanical ventilation (AOR 0.027; 95% CI 0.002–0.351; *p* = 0.006). Biomarkers did not achieve significance in the multivariate analysis.

## 4. Discussion

This study has revealed elevated circulating oxidants (MDA and ROS) and reduced antioxidants (CAT and GPx) in ICH patients compared to the healthy controls. Although the oxidant levels started declining after the 7th day, they remained elevated till day 15 compared to the healthy controls. Similarly, antioxidants (CAT and GPx) were reduced at admission and continued to improve but remained lower than those in the healthy controls even on day 15. Admission PHE correlated with day 1 ROS and GPx. Furthermore, day 1 hematoma volume, PHE, and HEC were significant predictors of CAT and MDA levels on day 15. High antioxidant levels on day 1 were associated with survival and good outcomes at one month. Overall, this study has comprehensively evaluated the temporal kinetics of circulating oxidative stress and antioxidant biomarkers in a large cohort of primary ICH patients and their association with clinical stroke severity, CT scan findings, and one-month outcomes.

There is a paucity of studies on circulating oxidative stress markers in ICH patients. The stress generated by the ICH and raised intracranial pressure is likely to increase catecholamine drive, leading to systemic oxidative stress through activation of circulating neutrophils and monocytes or macrophages, as well as the generation of more stable secondary oxidation products. Therefore, the observed changes in peripheral blood biomarkers are indirect indicators of the systemic response to ICH rather than a direct measure of oxidative stress within the brain [10,13,16,40]. Xia et al. studied MDA, SOD, IL-6, and TNF-α in serum and drain fluid obtained through minimally invasive drainage in the patients with hypertensive ICH and compared them with those treated conservatively. They reported reduced TNF-α, IL-6, and MDA levels and elevated SOD levels in the minimally invasive drainage group compared to the conservatively treated group. Additionally, there was improvement in NIHSS, hematoma volume, and HEC on the 7th, 14th, and 28th days in the minimally invasive drainage group [31]. Rendevski et al. studied CAT and advanced oxidation protein products, along with IL-6 and IL-10, in 73 conservatively treated ICH patients. However, there was no association between these biomarkers and PHE; rather, C-reactive protein (CRP), erythrocyte sedimentation rate (ESR), and neutrophil-to-lymphocyte ratio (NLR) predicted PHE [27]. Another study evaluated oxidative stress markers in the brain tissue overlying the hematoma and compared these with the brain tissue from an aneurysmal bleed or peritumor area. No significant difference was observed in protein oxidation, GSH, GPx, glutathione reductase, SOD, CAT, and total antioxidant status between the two groups [41]. This may be due to the inclusion of adjacent brain tissue from the aneurysm and tumor, as these areas may also have oxidative stress. In our study, we have observed elevated levels of oxidants and reduced levels of antioxidants in the ICH patients compared to the controls, which did not reach normal levels even on the 15th day. This may be attributed to the kinetics of hematoma resolution. The resolution of a hematoma depends upon its volume; smaller hematomas resolve within weeks, whereas larger hematomas may take months [42].

The majority of reported studies did not evaluate the role of day 1 oxidants and antioxidants on subsequent PHE. Perihematoma edema is a result of complex molecular and inflammatory cascades which develops at the beginning and increases after 72 h till 1 week [43]. Oxidative stress may induce both cytotoxic and vasogenic edema [44]. Cytotoxic edema induced by oxidative stress has been attributed to the destruction of ion transporters in the cell membrane, increased calcium influx, reduced ATP formation, activation of phospholipase, destruction of organelles and the cell membrane, and acidosis [45]. Free radicals stimulate nitric oxide (NO), matrix metalloproteinases (MMPs), and cytokines, thereby altering the blood–brain barrier and contributing to vasogenic edema [44]. Xia et al., demonstrated that improved PHE after minimally invasive craniopuncture is driven by the reduction of oxidative stress and inflammatory cytokines [31]. We noted a correlation between ROS and GPx and admission PHE. Day 1 volumes of ICH, PHE, and HEC also determined the antioxidant and oxidant status on day 15. Catalase and GPx resist oxidative stress by neutralizing free radicals [46]. The decrease in antioxidant status may be due to utilization and exhaustion. In a mouse model, treatment with exogenous GSH has shown neuronal protection and improvement [47]. Elevated CSF MDA and lower plasma total antioxidant status have also been reported an association with unfavorable outcomes in patients with ICH [28,29,32].

To reduce oxidative stress and enhance antioxidants, various strategies have been used in experimental studies, including deferoxamine (an iron chelator), statins (reduce inducible nitric oxide synthase), PPAR-γ (downregulates the tumor necrosis factor gene and nitric oxide synthase), minocycline (reduces malondialdehyde by inhibiting metalloproteinases and chelating iron), NXY-059 (scavenges ROS), edaravone (scavenges ROS), and targeted gene therapy against heme oxygenase-1 and heme oxygenase-2 (downregulates the rate-limiting enzyme for heme catabolism and iron production), and Nrf2 (upregulates the expression of a wide range of antioxidants) [10,48,49,50,51,52,53,54,55,56]. Glutathione peroxidase (GPx) is upregulated by baicalein, baicalin, curcumin, luteolin, quercetin, astragaloside IV, gastrodin, oleuropein, bakuchiol, isorhynchophylline, crocin, and chrysophanol in a rat model of ICH [26,57,58,59,60,61,62,63,64,65,66,67,68]. In a systematic review and meta-analysis including 38 randomized controlled trials involving 3454 patients, the role of edaravone administered within 7 days of ICH was evaluated. Edaravone could not reduce all-cause mortality or improve long-term functional outcomes [69]. However, edaravone was safe and reduced hematoma edema, NIHSS scores, and Barthel index scores [69,70]. The addition of both GPx enhancers and oxidant depletors for a period of 15 days or more may be helpful in the patients with ICH and warrants future studies.

In our study, the overall fatality rate at 1 month was 18.5%, and that in the study cohort was 17.2%. In a systematic analysis, the case fatality rate of ICH at one month ranged between 14% and 25% [71]. The strongest predictors of death in ICH are GCS score, NIHSS score, mechanical ventilation, hematoma volume, and HEC [7,72,73,74]. In the present study, the independent predictors of death were NIHSS score and mechanical ventilation.

## 5. Limitations

The ages of the patients and the volume of the hematoma were variable, which is inevitable in human studies. We did not measure oxidative and antioxidative stress biomarkers after day 15 of stroke. The observational design of this study limits causal interpretation, and the oxidative stress biomarkers were detected at predefined time points, which may not fully capture the temporal kinetics. Furthermore, other key factors contributing to PHE expansion, including inflammatory cytokines, ER stress, and metabolic factors, were not evaluated. These factors might also interact with oxidative stress signaling pathways. We used the microplate reader for oxidative biomarker quantification. Liquid chromatography–mass spectrometry (LC-MS) would have been a more sensitive method for quantification. Moreover, we measured circulating biomarkers, which provide indirect evidence of the systemic stress response following ICH. We did not assess inter-rater and intra-rater variability in volumetric CT scan measurements. Although magnetic resonance imaging (MRI) is the best method for measuring brain edema, we performed CT scans to reduce costs and image acquisition time. Cranial MRI requires at least 30 min, while a CT scan can be done in 5 min. Patients with ICH often have altered sensorium, hyperventilation, and extensor posturing. Therefore, subjecting them to MRI was not considered safe by the Ethics Committee.

## 6. Conclusions

Patients with ICH had higher oxidative stress and lower antioxidant levels, which remained abnormal until day 15. Oxidative stress biomarkers correlated with PHE, suggesting their role in the pathogenesis. Patients with higher levels of antioxidants had better survival and good outcome. Our findings provide an understanding of secondary brain damage and identify GPx as a potential therapeutic target. Future studies are required to evaluate the role of antioxidants in PHE and clinical outcomes, which will add further validity to the current findings and interpretations.

## Figures and Tables

**Table 1:** Cranial computerized tomographic scan findings.

Parameters	Day 1 (*n* = 87) [Median (Q1–Q3)]	Day 7 (*n* = 82) [Median (Q1–Q3)]	*p*-Value
Hematoma volume (mL)	25.0 (Q1, 19.0–Q3, 50.0)	25.0 (Q1, 16.0–Q3, 46.5)	0.85
Hematoma edema complex (mL)	30.0 (Q1, 22.0–Q3, 53.5)	31.0 (Q1, 21.0–Q3, 60.0)	0.16
Perihematoma edema (mL)	4.0 (Q1, 2.0–Q3, 7.0)	6.0 (Q1, 3.5–Q3, 9.5)	<0.001

**Table 2:** Biomarkers in the patients with intracerebral hemorrhage (ICH) and normal healthy control at different time points.

Parameters	Controls (*n* = 57), [Median (Q1–Q3)] (a)	ICH Patients [Median (Q1–Q3)]			*p*-Value					
		Day 1 (*n* = 87) (b)	Day 7 (*n* = 82) (c)	Day 15 (*n* = 78) (d)	Overall	a vs. b	b vs. c	c vs. d	b vs. d	a vs. d
ROS (FI)	4615.0 (Q1, 3125.5–Q3, 6134.7)	8541.5 (Q1, 5641.5–Q3, 14,886.0)	9171.2 (Q1, 5270.7–Q3 13,349.5)	5031.0 (Q1, 3549.0–Q3, 8516.5)	<0.001	<0.001	0.852	<0.001	0.014	0.144
MDA (nmol/L)	1.64 (Q1, 0.95–Q3, 3.19)	7.33 (Q1, 3.72–Q3, 9.24)	6.39 (Q1, 4.53–Q3, 8.25)	4.85 (Q1, 2.18–Q3, 6.36)	0.012	<0.001	0.387	<0.001	0.007	<0.001
Catalase (kU)	1.138 (Q1, 0.967–Q3, 1.364)	0.560 (Q1, 0.395–Q3, 0.792)	0.740 (Q1, 0.497–Q3, 0.981)	0.887 (Q1, 0.672–Q3, 1.094)	<0.001	<0.001	0.004	0.012	0.001	<0.001
GPx (nmol/GSH)	5.61 (Q1, 5.19–Q3, 5.86)	3.87 (Q1, 3.39–Q3 4.31)	4.23 (Q1, 3.89–Q3, 4.62)	4.45 (Q1, 3.93–Q3, 4.75)	0.002	< 0.001	0.011	0.167	0.001	<0.001

GPx = glutathione peroxidase; MDA = malondialdehyde; ROS = reactive oxygen species.

**Table 3:** Outcome of patients at one month.

Parameters (Day 1)	Good (mRS = 0–2) [*n* = 16 (18.4%)] Median (Q1–Q3) (a)	Poor (mRS = 3–5) [*n* = 56 (64.4%)] Median (Q1–Q3) (b)	Death (mRS = 6) [*n* = 15 (17.2%)] Median (Q1–Q3) (c)	*p*-Value			
				a vs. b	a vs. c	b vs. c	Overall
ROS (FI)	5889.5 (Q1, 4708.0–Q3, 16,665.0)	9270.5 (Q1, 6024.2–Q3, 14,967.2)	8710.5 (Q1, 4500.0–Q3, 12,625.3)	0.28	0.72	0.49	0.27
MDA (nmol/L)	6.9 (Q1, 5.24–Q3, 8.75)	7.63 (Q1, 3.55–Q3, 9.91)	7.17 (Q1, 3.42–Q3, 9.29)	0.63	1.00	0.84	0.52
Catalase (kU)	0.579 (Q1, 0.335–Q3, 0.771)	0.573 (Q1, 0.427–Q3, 0.862)	0.460 (Q1, 0.375–Q3, 0.560)	0.42	0.66	0.04	0.13
GPx (nmol/GSH)	3.99 (Q1, 3.05–Q3, 4.37)	3.93 (Q1, 3.55–Q3, 4.36)	3.30 (Q1, 2.62–Q3, 3.85)	0.88	0.10	0.008	0.03

GPx = glutathione peroxidase; MDA = malondialdehyde; mRS = modified Rankin Scale; ROS = reactive oxygen species.

**Table 4:** Predictors of death at one month in patients with intracerebral hemorrhage on univariate analysis.

Baseline Parameters	Survived [*n* = 72 (82.8%)] Median (Q1–Q3)	Death [*n* =15 (17.2%)] Median (Q1–Q3)	*p*-Value
Age (years)	59 (Q1, 50–Q3, 68)	54 (Q1, 48–Q3, 70)	0.67
Gender (Female) *	27 (31.0%)	6 (6.9%)	0.85
NIHSS score	16 (Q1, 11–Q3, 19)	27 (Q1, 25–Q3, 30)	<0.001
GCS score	13 (Q1, 11–Q3, 15)	7 (Q1, 6–Q3, 10)	<0.001
Antidiabetic drugs *	18 (25.0%)	1 (6.7%)	0.17
Antihypertensive drugs *	70 (97.2%)	14 (93.3%)	1.00
Anti-edema drugs *	44 (62.0%)	14 (93.3%)	0.003
Antiepileptic drugs *	19 (27.9%)	5 (33.3%)	0.76
Antibiotic drugs *	41 (58.6%)	13 (86.7%)	0.07
Mechanical ventilator *	5 (7.1%)	11 (73.3%)	<0.001
Hematoma volume (mL)	23.0 (Q1, 16.0–Q3, 39.0)	67.0 (Q1, 51.0–Q3, 98.0)	<0.001
HEC (mL)	28.5 (Q1, 21.0–Q3, 45.0)	67.5 (Q1, 38.0–Q3, 95.0)	<0.001
PHE (mL)	4.0 (Q1, 2.0–Q3, 7.0)	5.0 (Q1, 3.0–Q3, 10.0)	0.17
Serum creatinine (mg/dL)	1.15 (Q1, 0.96–Q3, 1.38)	1.36 (Q1, 1.00–Q3, 1.78)	0.11
Serum sodium (mmol/L)	138 (Q1, 134–Q3, 142)	141 (Q1, 132–Q3, 144)	0.48
Serum uric acid (mg/dL)	6.0 (Q1, 5.3–Q3, 7.8)	6.6 (Q1, 5.7–Q3, 7.8)	0.40
Serum albumin (g/dL)	4.11 (Q1, 3.70–Q3, 4.30)	4.00 (Q1, 3.66–Q3, 4.25)	0.45
Hemoglobin (g/dL)	13.3 (Q1, 12.5–Q3, 14.2)	13.4 (Q1, 11.7–Q3, 15.0)	0.63
ROS (FI)	8541.5 (Q1, 5700.2–Q3, 15,282.5)	8710.5 (Q1, 4500.0–Q3, 12,625.3)	0.65
MDA (nmol/L)	7.33 (Q1, 3.77–Q3, 9.16)	7.17 (Q1, 3.42–Q3, 9.29)	0.88
Catalase (kU)	0.575 (Q1, 0.411–Q3, 0.862)	0.460 (Q1, 0.375–Q3, 0.560)	0.07
GPx (nmol/GSH)	3.94 (Q1, 3.55–Q3, 4.36)	3.30 (Q1, 2.62–Q3, 3.85)	0.01

GCS = Glasgow Coma Scale, GPx = glutathione peroxidase; HEC = hematoma edema complex; MDA = malondialdehyde; NIHSS = National Institute of Health Stroke Scale; PHE = perihematoma edema; ROS = reactive oxygen species; * = *n* (%).

## Data Availability

The datasets generated during and/or analyzed during the current study are included in the main text, figures, tables, and Supplementary Materials. Further inquiries or additional datasets will be available from the first author and corresponding author upon request for scientific research purposes.

## Supplementary Materials

The following supporting information can be downloaded at: https://www.mdpi.com/article/10.3390/antiox15101260/s1, Table S1. Baseline characteristics of intracerebral hemorrhage patients. Table S2. Correlation biomarkers with volumetric parameters on computerized tomographic-scan.
