# Supplementary material for: Temporal Kinetics of Circulating Oxidative Stress Markers in Intracerebral Hemorrhage and Their Association with Perihematoma Edema
Source: Antioxidants (Basel). 2026 Oct 1;15(10):1260. doi: 10.3390/antiox15101260 (PMC13700692; doi:10.3390/antiox15101260)
Supplement: Supplementary file 1 [file antioxidants-15-01260-s001.zip › antioxidants-4477033-supplementary.pdf]

Supplementary Table S1. Baseline characteristics of intracerebral hemorrhage patients.

Baseline parameters		Number = 87 [n (%)]
Clinical findings		
Age in years*		58 (Q1, 50 – Q3, 68)
Gender (female)		33 (37.9%)
Risk factors	Hypertension	79 (90.8%)
	Diabetes mellites	19 (21.8%)
	Smoking	11 (12.6%)
	Tobacco chewers	29 (33.3%)
	Alcohol	19 (21.8%)
	Seizure	16 (18.4%)
	Hyperlipidaemia	53 (60.9%)
	Pneumonia	22 (25.3%)
Glasgow Coma Scale score *		12 (9 - 14)
NIHSS score *		18 (11 - 25)
Low density lipoprotein (mg/dL) *		96.0 (Q1, 73.0 – Q3, 128.0)
Serum creatinine (mg/dL) *		1.2 (Q1, 0.96 – Q3, 1.48)
Serum sodium (mmol/L) *		138.0 (Q1, 134.0 – Q3, 142.0)
Hemoglobin (g/dL) *		13.30 (Q1, 12.50 – Q3, 14.30)
Serum uric acid (mg/dL) *		6.0 (Q1, 5.30 – Q3, 7.80)
Systemic inflammatory response syndrome		66 (75.9%)
Intraventricular extension		39 (44.8%)
Pulse *		90 (Q1, 78 – Q3, 100)
Respiratory rate *		22 (Q1, 20 – Q3, 25)
Radiological findings		
ICH location	Lobar	16 (18.4%)
	Deep	71 (81.6%)
Hematoma volume (mL) *		25.0 (Q1, 19.0 – Q3, 50.0)
Hematoma edema complex volume (mL) *		30.0 (Q1, 22.0 – Q3, 53.5)
Absolute perihematoma edema volume (mL) *		4.0 (Q1, 2.0 – Q3, 7.0)

ICH = Intracerebral hemorrhage; NIHSS = National Institute of Health Stroke Scale
*-expressed in median (Q1 – Q3)

Supplementary Table S2. Correlation biomarkers with volumetric parameters on computerized tomographic-scan.

Parameters	Day 1 CT-scan			Day 7 CT-scan		
	Hematoma volume	HEC	PHE	Hematoma volume	HEC	PHE
Day 1						
ROS (FI)	r = -0.063<br>p = 0.562	r = 0.082<br>p = 0.509	r = 0.282<br>p = 0.024	r = 0.082<br>p = 0.504	r = 0.107<br>p = 0.400	r = 0.294<br>p = 0.019
MDA (nmole/L)	r = -0.003<br>p = 0.978	r = -0.220<br>p = 0.101	r = -0.025<br>p = 0.856	r = -0.209<br>p = 0.107	r = -0.191<br>p = 0.155	r = 0.071<br>p = 0.601
Catalase (kU)	r = -0.138<br>p = 0.207	r = 0.089<br>p = 0.490	r = -0.127<br>p = 0.325	r = 0.031<br>p = 0.806	r = 0.105<br>p = 0.415	r = 0.238<br>p = 0.063
GPx (nmole/GSH)	r = -0.148<br>p = 0.179	r = -0.116<br>p = 0.373	r = -0.290<br>p = 0.023	r = -0.083<br>p = 0.513	r = -0.130<br>p = 0.318	r = -0.230<br>p = 0.075
Day 7						
ROS (FI)	r = 0.026<br>p = 0.819	r = 0.100<br>p = 0.447	r = 0.149<br>p = 0.255	r = 0.167<br>p = 0.187	r = 0.185<br>p = 0.157	r = 0.359<br>p = 0.005
MDA (nmole/L)	r = 0.195<br>p = 0.126	r = 0.121<br>p = 0.433	r = 0.049<br>p = 0.752	r = 0.183<br>p = 0.217	r = 0.138<br>p = 0.373	r = 0.067<br>p = 0.665
Catalase (kU)	r = - 0.195<br>p = 0.106	r = - 0.224<br>p = 0.106	r = - 0.090<br>p = 0.522	r = -0.196<br>p = 0.144	r = -0.208<br>p = 0.135	r = -0.143<br>p = 0.307
GPx (nmole/GSH)	r = 0.197<br>p = 0.114	r = 0.244<br>p = 0.088	r = - 0.023<br>p = 0.872	r = 0.247<br>p = 0.072	r = 0.255<br>p = 0.074	r = 0.136<br>p = 0.348
Day 15						
ROS (FI)	r = 0.142<br>p = 0.267	r = 0.060<br>p = 0.673	r = 0.125<br>p = 0.377	r = 0.157<br>p =0.257	r = 0.163<br>p = 0.248	r = 0.340<br>p = 0.014
MDA (nmole/L)	r = 0.162<br>p = 0.312	r = 0.237<br>p = 0.208	r = 0.379<br>p = 0.039	r = 0.301<br>p = 0.094	r = 0.309<br>p = 0.096	r = 0.532<br>p = 0.002
Catalase (kU)	r = - 0.323<br>p = 0.017	r = -0.370<br>p = 0.019	r = -0.429<br>p = 0.006	r = - 0.311<br>p = 0.043	r = - 0.369<br>p = 0.019	r = - 0.441<br>p = 0.004
GPx (nmole/GSH)	r = - 0.050<br>p = 0.740	r = 0.003<br>p = 0.987	r = -0.075<br>p = 0.664	r = - 0.064<br>p = 0.697	r = - 0.005<br>p = 0.979	r = - 0.003<br>p = 0.984

HEC = hematoma edema complex; GPx = glutathione peroxidase; MDA = malonaldehyde; PHE = perihematoma edema; ROS = reactive oxygen species.
